# Global Proteomic Analysis Reveals Widespread Lysine Succinylation in Rice Seedlings

**DOI:** 10.3390/ijms20235911

**Published:** 2019-11-25

**Authors:** Kai Zhang, Yehui Xiong, Wenxian Sun, Guo-Liang Wang, Wende Liu

**Affiliations:** 1 State Key Laboratory for Biology of Plant Diseases and Insect Pests, Institute of Plant Protection, Chinese Academy of Agricultural Sciences, Beijing 100193, China; kaisero@163.com (K.Z.); yhxiong0706@163.com (Y.X.);; 2 College of Plant Protection, China Agricultural University, Beijing 100193, China; 3 School of Life Sciences, Tsinghua University, Beijing 100084, China; 4 The Ohio State University, Columbus, OH 43210, USA

**Keywords:** lysine succinylation, post-translational modification, rice seedlings

## Abstract

Lysine succinylation (Ksu) is a dynamic and reversible post-translational modification that plays an important role in many biological processes. Although recent research has analyzed Ksu plant proteomes, little is known about the scope and cellular distribution of Ksu in rice seedlings. Here, we report high-quality proteome-scale Ksu data for rice seedlings. A total of 710 Ksu sites in 346 proteins with diverse biological functions and subcellular localizations were identified in rice samples. About 54% of the sites were predicted to be localized in the chloroplast. Six putative succinylation motifs were detected. Comparative analysis with succinylation data revealed that arginine (R), located downstream of Ksu sites, is the most conserved amino acid surrounding the succinylated lysine. KEGG pathway category enrichment analysis indicated that carbon metabolism, tricarboxylic acid cycle (TCA) cycle, oxidative phosphorylation, photosynthesis, and glyoxylate and dicarboxylate metabolism pathways were significantly enriched. Additionally, we compared published Ksu data from rice embryos with our data from rice seedlings and found conserved Ksu sites between the two rice tissues. Our in-depth survey of Ksu in rice seedlings provides the foundation for further understanding the biological function of lysine-succinylated proteins in rice growth and development.

## 1. Introduction

Post-translational modifications (PTMs) of proteins are one of the most important biological processes for expanding the genetic code and regulating cellular physiology [1,2]. Lysine, one of the three basic amino acid residues crucial for protein spatial structure and function [3], can be subjected to multiple PTMs, including ubiquitination [4], methylation [5,6], acetylation [7,8], and succinylation [3]. Lysine succinylation is defined as the transfer of a succinyl group to a lysine residue [9], which can alter the charge of the lysine residue under certain physiological pH conditions. Compared with other PTMs, lysine succinylation induces more substantial changes to a protein’s chemical properties than either methylation or acetylation, both of which are important for cellular processes [3]. First identified in *Escherichia coli* [3], lysine succinylation has been found in many other species. Succinylome analysis has been performed on *Saccharomyces cerevisiae*, *Homo sapiens* Hela cells, *Mus mucsculus* liver tissue [10], *Mycobacterium tuberculosis* [11], and *Toxoplasma gondii* [12]. Increasing evidence suggests that lysine succinylation is an evolutionarily widespread and conserved modification in eukaryotes and prokaryotes [9,11,13]. Furthermore, succinylated lysine can be desuccinylated [14,15]. Park et al. reported that SIRT5 can remove malonyl and succinyl moieties from target lysine residues [15]. Lin et al. also found that SIRT5 can bind to, desuccynylate, and activate SOD1 [14]. These results show that lysine succinylation (Ksu) is a dynamic and reversible PTM.

Rice is one of the most important crops for humans, providing staple food to more than half the world population [16]. It has also become the monocot plant model for plant breeding and biological research, after several cultivated and wild rice lines were re-sequenced and the functional genes identified [17]. Many traditional proteomic studies have broadened our understanding of rice biological processes. For example, a two-dimension(DE) map comparison showed 148 differentially expressed proteins in the germination process of rice seeds [18], and a total of 563 differentially expressed proteins were identified in rice hull development using Isobaric Tag for Relative Absolute Quantitation(iTRAQ) MS/MS [19]. Furthermore, studies have focused on proteome reprogramming following rice treatment. For example, a total of 29 unique methyl-jasmonate (MeJa)-related proteins were identified in rice, many of which were associated with reactive oxygen species (ROS) accumulation and plant defense response [20], and the proteome reprogramming of plants treated with MeJa induced defense responses against wounding [21]. Haynes et al. identified 236 cold-responsive proteins and 85 proteins with the iTRAQ approach in rice seedlings [22]. When compared with transcription and translation, PTMs could help trigger a much faster response that ensures that plant cells can adapt to environmental changes. Recent advances in proteomic technology have improved the analysis of the global succinylome and the identification of Ksu. For example, 699 lysine-acetylated sites on 389 proteins and 665 lysine-succinylated sites on 261 proteins were identified in rice embryos, including both acetylation and succinylation on individual proteins [23]. In rice seedlings, 1337 lysine acetylation (Kac) sites and 716 Kac proteins were identified [24], but no proteome-wide Ksu sites were reported. A systematic analysis of succinylation in rice seedlings will provide greater insights into rice growth and development.

In this study, we performed a global analysis of lysine succinylation in rice seedlings (cultivar Nipponbare). We identified 710 Ksu sites on 346 Ksu proteins involved in diverse biological functions and localized in various subcellular compartments. Bioinformatic analyses found six unique motifs in the sequences flanking the succinylation sites and that succinylation modulated a wide range of biological processes in rice. Compared with published data [24,25], we report detailed crosstalk information between the reversible Kac and Ksu during the rice seedling stage and the dynamic change of PTMs between the embryo and the seedling stage. These results will facilitate future biological analyses of succinylation in rice or other plants.

## 2. Results

### 2.1. Global Analysis of Ksu Sites in Rice

**Figure 1:**
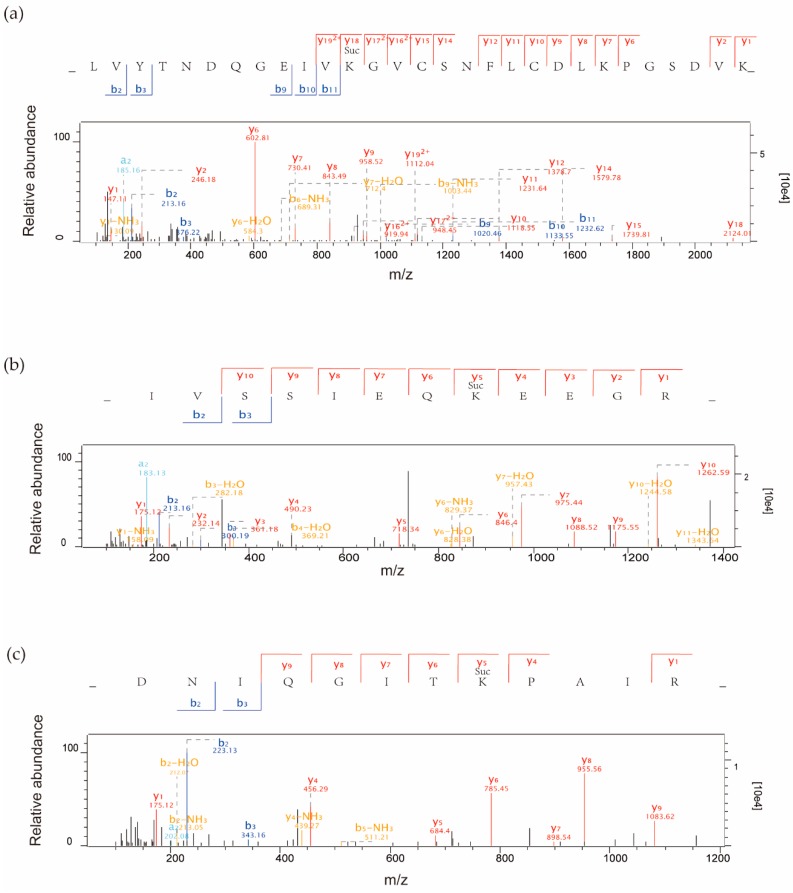
Representative MS/MS spectra of succinylpetides from three proteins: (**a**) succinylated peptide _LVYTNDQGEIVK(su)GVCSNFLCDLKPGSDVK_ with a succinylated site at K177 from the chloroplast protein petH-ferredoxin-NADP(+) reductase (Q0DF89); (**b**) succinylated peptide _IVSSIEQK(su)EEGR_ with a succinylated site at K70 from the 14-3-3-like protein GF14c (Q6ZKC0); and (**c**) succinylated peptide _DNIQGITK(su)PAIR_ with a succinylated site at K31 from histone H4(Q7XUC9).

Lysine succinylation, important in regulating protein function in both prokaryotic and eukaryotic cells, is emerging as a new protein PTM [10]. Here, 710 succinylation sites in 346 proteins were identified using affinity enrichment and LC–MS/MS as follows (Figure S1a). Firstly, we checked the mass error of all the identified peptides. The distribution of mass error was near zero and for most peptides, it was less than 0.02 Da, indicating that the mass accuracy of the MS data fitted the requirement (Figure S1b). Secondly, the length of most peptides was between 8 and 20 amino acids, which agrees with the features of tryptic peptides (Figure S1c). To further understand the functions and features of these identified proteins, we annotated them considering several different categories, including gene ontology (GO), protein domain, KEGG Pathway, and subcellular localization; all detailed data are listed in Supplementary Table S1. Three MS/MS spectra of succinylated peptides are shown in Figure 1.

### 2.2. Analysis of Succinylated Lysine Motifs

**Figure 2:**
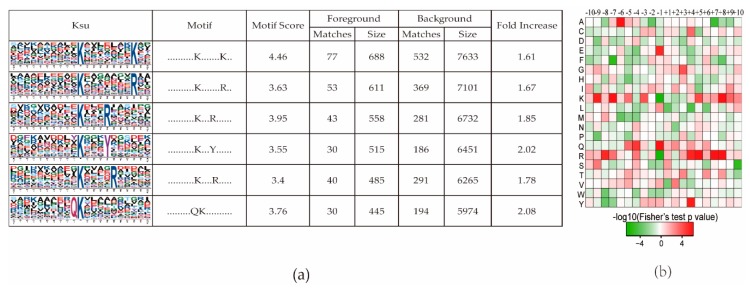
Motif analysis of all identified lysine succinylation (Ksu) sites. (**a**) Succinylation motifs and conservation of succinylation sites. The size of each letter corresponds to the frequency of that amino acid residue. (**b**) Heat map of the amino acid compositions of the succinylated sites showing the frequency of different amino acids surrounding the succinylated lysine.

To determine the conserved motifs surrounding the succinylated lysine, the software Motif-X was used to analyze the conserved amino acids in all of the identified Ksu proteins. Six conserved motifs were identified in this study (Figure 2a). Arginine (R) was the most conserved amino acid surrounding a succinylated lysine (Figure 2b); three R residues were found downstream of Ksu conserved motifs (Figure 2a) at the +4, +5, and +8 positions. The other three conserved amino acids surrounding a succinylated lysine were lysine (K), glutamine (Q), and tyrosine (Y) (Figure 2a).

### 2.3. GO Classification of Succinylated Proteins

**Figure 3:**
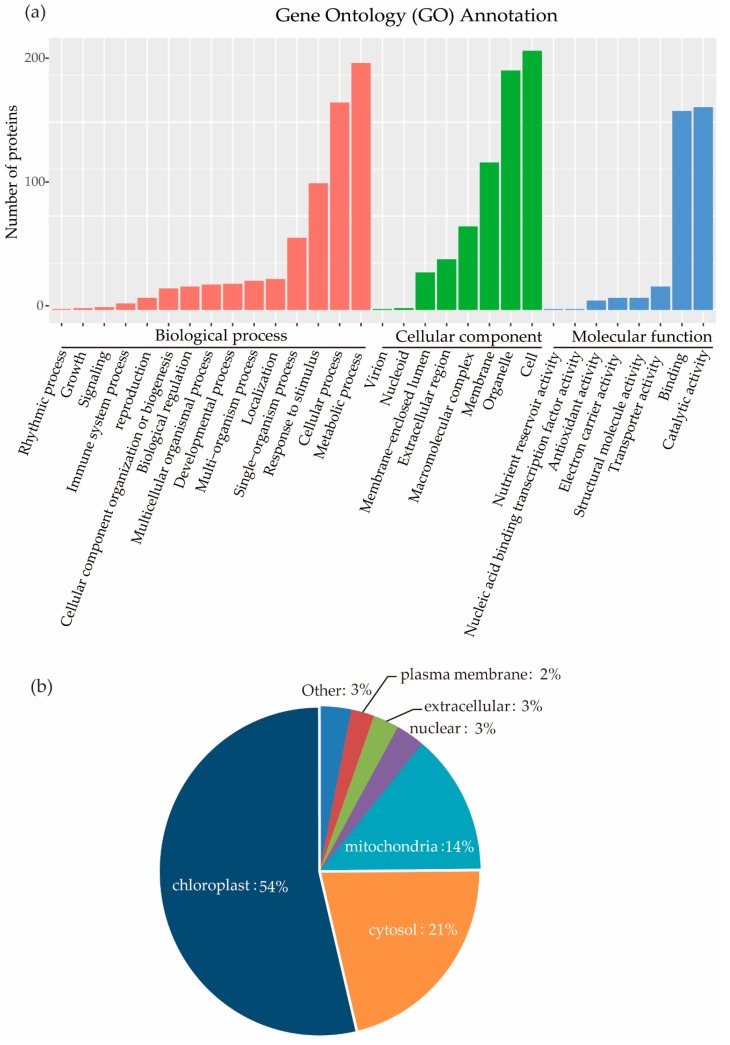
(**a**) GO Classification of succinylated proteins based on second-level terminology. (**b**) Subcellular localization of the identified succinylated proteins from GO analysis.

To better understand the cellular functions of succinylated proteins in rice, GO functional classification of all the identified proteins was carried out according to the second-level terminology of biological processes, molecular functions, and cellular components (Figure 3a, Supplementary Table S2). For biological processes, the largest group included 263 succinylated proteins that were involved in metabolic processes, follow by 221 Ksu proteins that participated in cellular processes. For molecular functions, almost half of the proteins (216) had catalytic activity, while the other half (212) had binding activity. If we classified proteins as cellular components, 255 proteins were localized in organelles, and 157 in a membrane.

### 2.4. Distribution of Ksu Proteins in Subcellular Compartments

Most proteins localize in their appropriate subcellular compartments to perform their function. To understand the distribution of succinylation proteins, we analyzed the subcellular localization of all 346 proteins identified. Over half of the identified proteins (54%) were predicted to be localized in chloroplast, 21% in the cytosol, 14% in the mitochondria, and only a few proteins in the extracellular space (3%), nucleus (3%), and plasma membrane (2%) (Figure 3b).

### 2.5. Domain Enrichment of Succinylated Proteins in Rice

**Figure 4:**
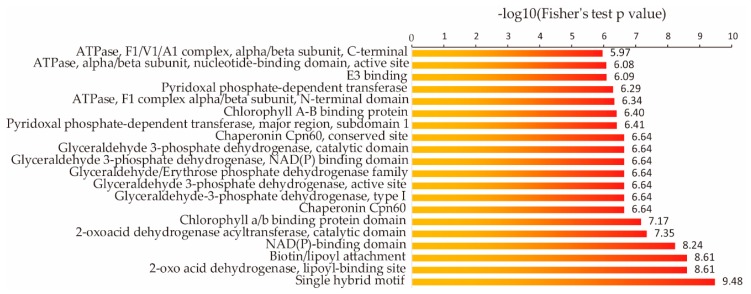
Protein domain enrichment analysis of succinylated proteins in rice seedlings.

We speculated that some specific proteins might be enriched in the rice succinylome. We conducted a protein domain enrichment analysis of the succinylome (Figure 4) and found that 20 protein domains were significantly enriched in it, including the single hybrid motif, 2-oxo acid dehydrogenase, lipoyl-binding site, biotin/lipoyl attachment domain, and NAD(P)-binding domain.

### 2.6. Protein Succinylation Regulates Diverse Metabolic Pathways in Rice

**Figure 5:**
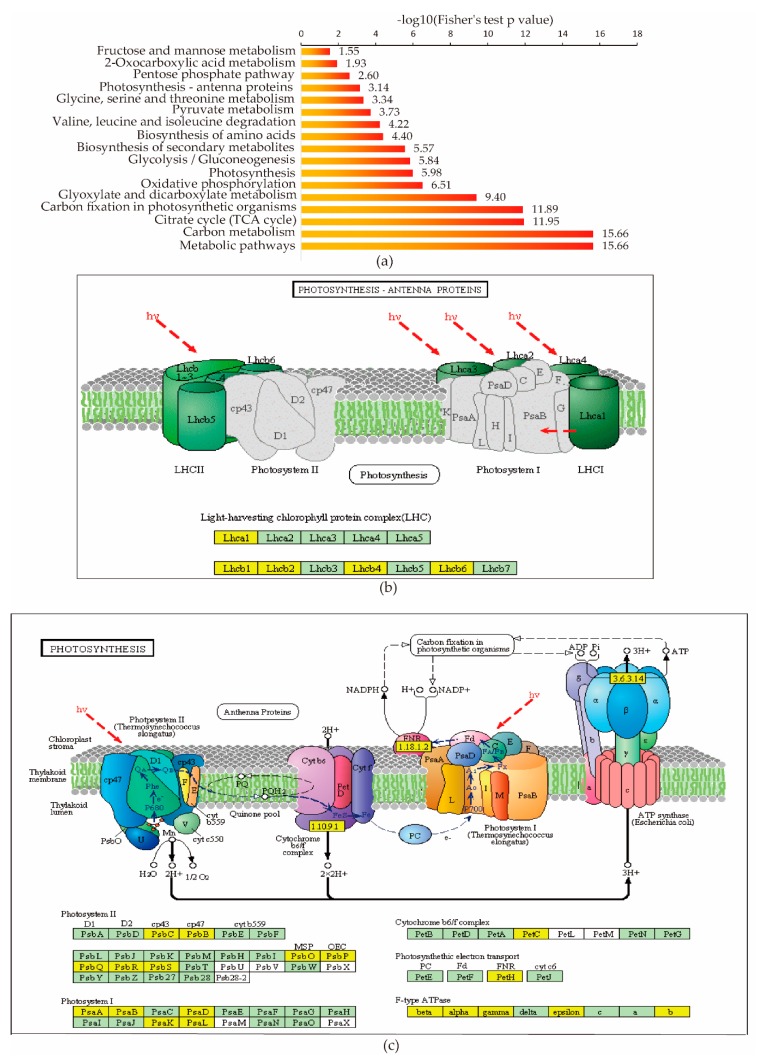
Protein succinylation regulates diverse metabolic pathways in rice. (**a**) KEGG pathway-based enrichment analysis of the identified proteins. (**b**,**c**) Key enzyme with succinyl post-translational modifications (PTM) in photosynthesis pathways.

To determine which types of pathways are preferred lysine succinylation targets, a pathway enrichment analysis was performed. The results showed that protein succinylation was involved in multiple metabolic pathways, including carbon metabolism, citric acid cycle (TCA cycle), oxidative phosphorylation, photosynthesis, and glyoxylate and dicarboxylate metabolism (Figure 5a and Supplementary Table S3). These results suggest that protein succinylation in rice seedlings regulates diverse metabolic pathways.

Plant proteins involved in photosynthesis are important to plant growth and development. We found succinylation present in many protein subunits related to photosynthesis, such as Lhca1, Lhcb1, Lhcb2, Lhcb4, and Lhcb6 in the light-harvesting chlorophyll protein complex (LHC) (Figure 5b), as well as PsbC, PsbB, PsbO, PsbP, PsbQ, PsbR, PsbS in photosystem II, PsaA, PsaB, PsaD, PsaK, PsaL in photosystem I, PetC in cytochrome b6/f complex, and PetH in photosysthetic electron transport and beta, alpha, gamma, epsilon F-type ATPase (Figure 5c). Lysine succinylation is also involved in carbon metabolism, including carbon fixation. We found lysine succinylation occurred in nearly every branch of the Calvin cycle: many proteins, like fructose-1,6-bisphosphatase, phosphoglycerate kinase, and phosphoribulokinase, were identified as succinylated proteins.

### 2.7. Lysine Succinylation in Histone Protein

Succinylation of histone proteins has been reported in many species, including yeast, bacteria, animals, tomato, and rice. In our study, we found that succinylation modification occurred on histones H3K23, H3K56, H3K79, H4K31. Succinylation of H3K56 was previously found in *T. gondii*, *Drosophila melanogaster*, *M. musculus* and *H. Sapiens* [12]. H3K79 and H4K31 were also found in *D. melanogaster*, *M. musculus*, *H. sapiens*, *S. cerevisiae*, and *Solanum lycopersicum* [26], suggesting that these succinylation sites are conserved in eukaryotes (Figure S2). Many different PTMs have been reported in H3K23, including propionylation, ubiquitylation, and methylation, but no succinylation was found [27]. In the present study, we discovered that H3K23 can be succinylated. Although the function of succinylation in histone is not clearly understood, succinylation of H3K23 may be important for histone structure and function.

### 2.8. Comparing Lysine Succinylated Proteins in Rice Embryo and Seedlings

Recently, a global proteome analysis of lysine acetylation and succinylation in the embryo of rice seed was reported [23]: 665 lysine succinylated sites were identified in 261 proteins. The succinylated proteins were found localized in the cytosol (36%), chloroplast (30%), mitochondria (13%), and nucleus (10%; Figure S3). In our study, we found more succinylated protein localized in rice seedling chloroplasts (54%; Figure 3b) compared with embryos. This is understandable, as rice seedling development requires more chloroplast proteins for photosynthesis.

**Figure 6:**
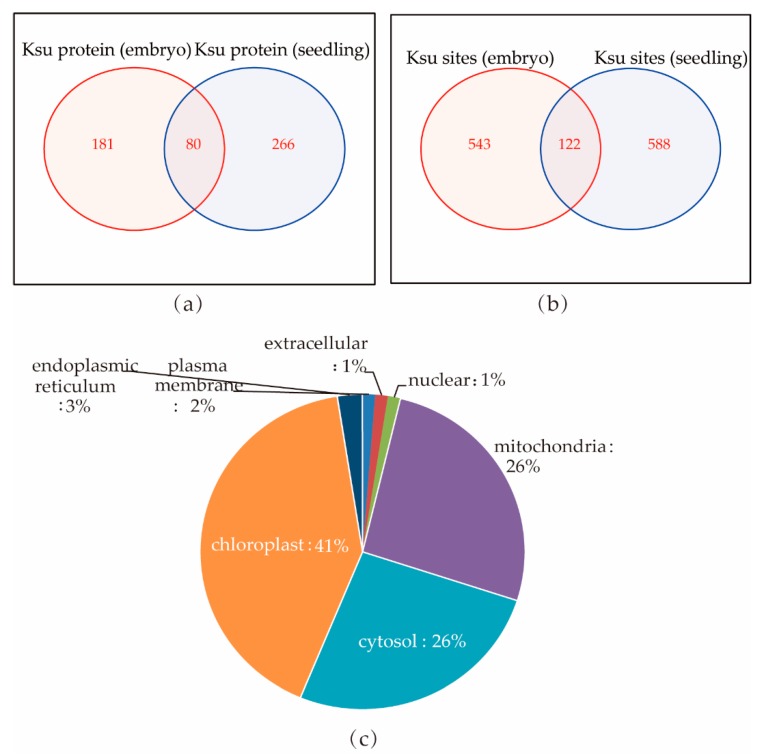
Comparing lysine succinylated proteins between rice embryo and seedling. (**a**,**b**) Venn diagram of succinylated proteins and sites that overlapped in embryos and seedlings; 80 protein contained Ksu sites in both embryos and seedlings, and only 122 Ksu sites had conserved motifs. (**c**) Subcellular localization of the overlapping succinylated and acetylated proteins in rice embryos and seedlings.

We then compared the individual succinylated proteins between rice seedlings and embryos. We found only 80 proteins—including Q9AUV6 (UDP-glucose 6-dehydrogenase 3), Q69QQ6 (heat shock protein 81-2), Q53LQ0 (endosperm storage protein 2), and Q6ZKC0 (14-3-3-like protein GF14c)—that were succinylated in both rice embryos and seedlings. Within these 80 proteins, 122 succinylated sites from 67 proteins overlapped (Figure 6a,b; Supplement Tables S4 and S5). Most of the overlapping succinylated proteins were localized in the chloroplast (41%), cytosol (26%), and mitochondria (26%; Figure 6c). Half of these overlapping proteins are involved in different metabolic pathways. For examples, succinate-semialdehyde dehydrogenase B9F3B6, isocitrate dehydrogenase Q9SDG5 and Q7XMA0, and glyceraldehyde 3-phosphate dehydrogenase Q0J8A4 and Q7FAH2 participate in carbon metabolism, Q9FRX7 which is an aldehyde dehydrogenase, participates in glycolysis, and nucleoside diphosphate kinase Q5TKF4 participates in purine metabolism. UTP–glucose-1-phosphate uridylyltransferase Q93X08, UDPglucose 6-dehydrogenase Q9AUV6, and phosphoglucomutase Q9AUQ4 participate in starch and sucrose metabolism.

### 2.9. Alternative Lysine Succinylation and Acetylation in Rice Seedlings

**Figure 7:**
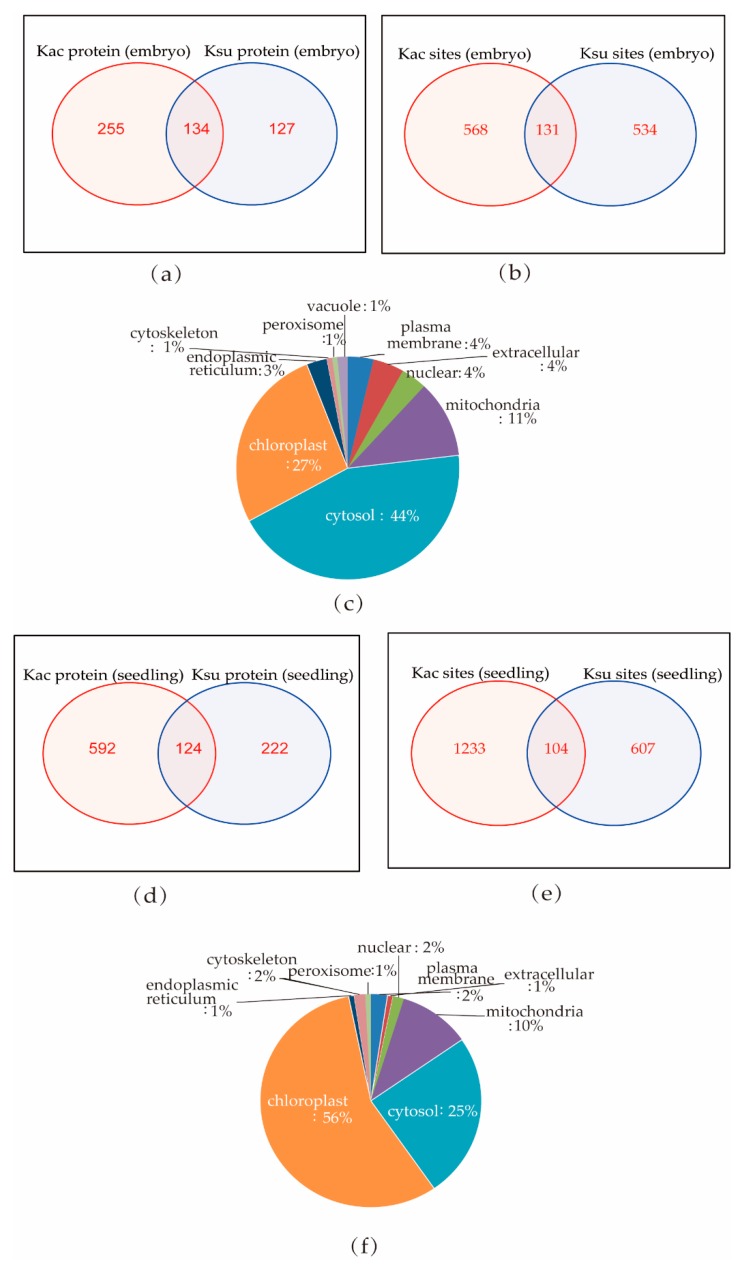
Lysine succinylation and acetylation of individual protein sites in rice: (**a**,**b**) Venn diagram of overlapping PTM proteins and sites in rice embryos; 134 proteins contained both lysine acetylation (Kac) and Ksu sites in the embryo, and 131 sites overlapped. (**c**) Subcellular localization of overlapping succinylated and acetylated proteins in rice emybros. (**d**,**e**) Venn diagram of overlapping PTM proteins and sites in rice seedling; 124 proteins contained both Kac and Ksu sites, and 104 sites overlapped. (**f**) Subcellular localization of overlapping succinylated and acetylated proteins in rice seedling.

It has been reported that many PTMs can occur at the same protein motifs [23,28]. We found that 134 proteins could be both acetylated and succinylated in embryos, and 131 sites in 73 proteins overlapped (Figure 7a,b; Supplementary Tables S4 and S5). We compared our current succinylome data with the acetylome data collected from our previous study that analyzed lysine acetylation in rice seedlings [24] and found that 124 proteins could be both acetylated and succinylated (Figure 7d,e), while only 104 sites in 68 proteins overlapped.

The overlapping proteins in rice embryos and seedlings were found widely distributed in the cytosol, mitochondrial, nuclear, plasma membrane, peroxisome, and chloroplast (Figure 7c,f). In rice seedlings, more overlapping PTM proteins were localized in the chloroplast (56%), and fewer in the cytosol (25%), while rice embryos had 27% overlapping PTM in the chloroplast and 44% in the cytosol. This indicates a dynamic competition between acetylation and succinylation at different developmental stages of rice. Histone H3 (Q2RAD9) and Histone H4 (Q7XUC9) were both succinylated and acetylated in rice seedlings, while Histone H4 could only be acetylated in rice embryos. These results suggest that specific regulation of the two PTMs, acetylation and succinylation, exists in histones and may contribute to the regulation of gene transcription. Only 29 of the overlapping PTM proteins were found in both rice embryos and seedlings, and only 9 modified sites in 9 proteins could be both succinylated and acetylated (Figure S4a,b; Supplementary Tables S4 and S5). The nine proteins were Q0IXR7, Q43008, Q5N725, Q6F361, Q6ZH98, Q7XXS0, Q9ASP4, Q9AUQ4, and Q9SXP2. Interestingly, these proteins were annotated as different kinds of enzymes found in the cytosol, mitochondria, and chloroplast.

## 3. Discussion

We have here provided a comprehensive analysis of the succinylome in rice (*Oryza sativa*. cultivar Nipponbare) seedlings. A total of 710 succinylation sites in 346 proteins were identified (Figure S1a). Six putative succinylation motifs were identified, and the succinylation site was found to be surrounded by arginine (R), lysine (K), or tyrosine (Y) in the downstream direction or by glutamine (Q) in the upstream direction (Figure 2a). Analyzing the subcellular localization and molecular function of lysine succinylation revealed that the lysine-succinylated proteins were distributed across diverse cellular compartments involved in many important biological functions, especially photosynthesis. A previous report identified 665 succinylated sites in 261 proteins in rice seeds (*O.sativa* cultivar Nipponbare) [23], and 131 of these Ksu proteins were present in our data set. Another report identified 2593 succinylated proteins in rice leaves, but a different cultivar was used, Wuyunjing 7 [29]. These studies used approaches similar to ours, so the variations in the results may be due to the different growth stages and varieties of rice analyzed. Our study presents new knowledge of the roles of protein lysine succinylation in the growth and development of rice plants. Furthermore, our approach might be applied to comprehensively understand the lysine succinylation landscape in other plants.

Arginine (R) is the conserved amino acid surrounding the Ksu sites; this was reported also in other species, such as the marine bacterium *Vibrio parahaemolyticus* [30]. Arginine has the highest ratio of nitrogen to carbon among all the 21 amino acids. Since arginine methylation is a prevalent PTM found in both nuclear and cytoplasmic proteins [31], it is likely that some of the arginine residues surrounding the succinylated lysine are potential targets of other PTM enzymes, such as protein arginine methyltransferases. The methylation of arginine residues affects diverse cellular processes, such as signal transduction and mRNA splicing in rice [32]. Whether the arginine residues surrounded the Ksu sites have similar functions remains to be confirmed. We also found that lysine (K) and glutamine (Q) surrounded Ksu sites in rice seedlings, which is consistent with observations in *T.gondii* [12] and drug-resistant *M. tuberculosis* [11]. These results indicate that different species may share conserved motifs surrounding a succinylated lysine, even though the motif positions may be different.

GO classification of rice plants found substantial succinylation in enzymatic proteins involved in catalytic progress and in binding proteins involved in protein–protein interaction or DNA transcription (Figure 3a). Ksu protein distribution in rice seedlings showed that succinylation was prevalent in proteins involved in photosynthesis, protein transport, and respiration (Figure 3b).

Among the 20 significantly enriched domains we identified in this study (Figure 4), a single hybrid motif was found in the biotinyl/lipoyl carrier protein; biotin/lipoic acid acts as a covalent cofactor in enzymes that catalyze metabolic reactions. This was also observed in acetyl-CoA carboxylase from *E. coli* [33] and in protein H of the glycine cleavage system in *Pisum sativum* [34]. We observed that glyceraldehyde-3-phosphate dehydrogenase (GAPDH) was the enzyme with the highest number of NAD(P)-binding domains. GAPDH is essential for maintaining cellular ATP levels and carbohydrate metabolism [35]. GAPDH proteins are acetylated in animals [36], *Arabidopsis* [37], and rice [38]. The acetylated form of GAPDH in the nucleus supposedly functions as a transcriptional activator that stimulates glycolysis [38], and it is possible that succinylated GAPDH similarly regulates glycolysis. These results indicate that succinylated proteins with enriched domains are important for various cellular functions in rice.

Pathway enrichment analysis revealed that most succinyl-CoA synthesis and associated enzymes from the tricarboxylic acid cycle (TCA), such as succinyl-CoA ligase beta subunit (Q6K9N6) and dihydrolipoamide succinyltransferase (Q7XVM2), were succinylated (Figure 5a, Supplementary Table S3). Succinyl-CoA is an important enzyme cofactor regulating succinylation and an intermediate metabolite of TCA [39]. TCA is the major carbon metabolic pathway in plants that supplies electrons during oxidative phosphorylation within the inner mitochondrial membrane. These findings suggest that lysine succinylation might be important for carbon metabolism and regulation of photosynthesis by modifying different chloroplast proteins.

Rice seeds accumulate large quantities of storage proteins during germination and seedling growth to serve as nitrogen sources. We found many Ksu sites in Q53LQ0 (endosperm storage protein 2) in rice seeds (K26, K88, K111, K246, K368) and rice seedlings (K74, K88, K128, K134, K147). Interestingly, K88 was succinylated in both developmental stages (Supplement Tables S4 and S5). Successfully trafficking storage proteins is very important for sustaining plants and animals. Wang et al. [40] showed that proteins such as OsRab5a regulate storage proteins trafficking in rice endosperm cells, but no reports showed that PTMs could also regulate storage proteins trafficking. As embryos often differentiate into unique seedlings, we assume that different Ksu sites regulate storage proteins trafficking.

Our analysis also showed that K70 succinylation from the 14-3-3-like protein GF14c is conserved in both rice embryos and seedlings (Supplement Tables S4 and S5). The 14-3-3-like protein GF14c can interact with florigen Hd3a and act as a negative regulator of flowering in rice [41]. This indicates that lysine succinylation in GF14c may regulate rice flowering. In addition, the comparison between embryos and seedlings of rice provides more insights into the metabolic role of protein succinylation.

We identified nine proteins (Q0IXR7, Q43008, Q5N725, Q6F361, Q6ZH98, Q7XXS0, Q9ASP4, Q9AUQ4, and Q9SXP2) that conserved lysine acetylation and succinylation motfis in both the embryo and the seedling stage (Supplement Tables S4 and S5). Ribosomal protein S28e (Q0IXR7) is a precursor to RNA splicing and mRNA maturation [42]. Q43008 is a superoxide dismutase (SOD) in mitochondria that can decrease SOD activity when succinylated in humans [14]. Fructose-bisphosphate aldolase is a key enzyme in the glycolysis pathway [43]. The competition between the two PTMs in fructose-bisphosphate aldolase 3 (Q5N725) indicate a potential function of lysine succinylation and acetylation in regulating glycolytic enzyme activities. Q6F361 is a malate dehydrogenase that has been reported to be negatively regulated during rice stress response to salt [44]. Q6ZH98 is a cyclophilin-type peptidyl-prolyl cis-trans isomerase associated with the photosynthetic membranes in *Arabidopsis* [45]. Q7XXS0 is subunit D of F_0_ ATPase. F_0_F_1_-ATPase synthesizes ATP during oxidative phosphorylation [46]. Q9ASP4 is a putative dihydrolipoamide dehydrogenase in rice. Dihydrolipoamide dehydrogenase is one of three enzymes in the pyruvate dehydrogenase complex (PDC) that converts pyruvate into acetyl-CoA [47]. Q9AUQ4 is a putative α-d-phosphohexomutase with reportedly diverse roles in carbohydrate metabolism, from bacteria to humans [48]. Q9SXP2 is a d-isomer specific 2-hydroxyacid dehydrogenase with a NAD-binding domain. NAD-dependent hydroxyacid dehydrogenase is important for carbohydrate metabolism [49]. These results indicate that alternative lysine succinylation and acetylation exists in many proteins from diverse pathways in rice. We hypothesize that lysine succinylation and acetylation can dynamically regulate different pathway—such as carbohydrate metabolism, glycolysis pathway, and photosynthetic pathway—to fulfill plant development at different stages.

## 4. Materials and Methods

### 4.1. Rice Plants and Growth Conditions

Rice (*Or. sativa*, cultivar Nipponbare) seeds were surface-sterilized with 10% (*w*/*v*) NaClO for 20 min, washed extensively with distilled water, and then germinated in half-strength Murashige and Skoog medium [50] at 28 °C for 7 days. Three biological replicates were obtained in this experiments. Ten rice seedlings of each replicate were transplanted into soil and grown in a growth chamber with a 16 h-day/8 h-night cycle and 80% relative humidity. After five weeks of growth, the whole rice plants, including leaves, stems, and roots, were washed and harvested for protein extraction.

### 4.2. Protein Extraction and Trypsin Digestion

Samples were initially ground with liquid nitrogen before transferring the cell powder to a 5 mL centrifuge tube and sonicating three times on ice using a high-intensity ultrasonic processor (Scientz Corporation, Ningbo, China) in lysis buffer (8 M urea, 1% (*v*/*v*) Triton-100, 10 mM DTT, 2 mM EDTA, 3 µM Trichostatin A (TSA), 50 mM nicotinamide (NAM), and 0.1% (*w*/*v*) Protease Inhibitor Cocktail IV). The remaining debris were removed by centrifugation at 20,000 *g* at 4 °C for 10 min. The proteins were precipitated with cold 15% TCA for 2 h at −20 °C. After centrifugation at 4 °C for 10 min, the supernatant was discarded. The remaining precipitate was washed three times with cold acetone. The proteins were re-dissolved in buffer (8 M urea, 100 mM NH_4_CO_3_, pH 8.0), and the protein concentration was measured according to the 2-D Quant kit instructions (GE Healthcare Company, Arlington Heights, IL, USA).

For protein digestion, the protein solution was reduced with 10 mM DTT for 1 h at 37 °C and alkylated with 20 mM indoleacetic acid (IAA) for 45 min at room temperature in darkness. For trypsin digestion, the protein sample was diluted by adding 100 mM NH_4_CO_3_ to urea at less than 2 M concentration. Trypsin was then added at a 1:50 trypsin-to-protein mass ratio for the first digestion overnight and at a1:100 trypsin-to-protein mass ratio for a second 4 h digestion.

### 4.3. High-Performance Liquid Chromatography (HPLC) Fractionation and Affinity Enrichment

The sample was then fractionated by high-pH reverse-phase HPLC using Agilent 300Extend C18 (Agilent Corporation, Santa Clara, CA, USA) column (5 µm particles, 4.6 mm ID, 250 mm length). Peptides were first separated with a gradient of 2% to 60% acetonitrile (ACN) in 10 mM ammonium bicarbonate pH 10 for 80 min, obtaining 80 fractions. Then, the peptides were combined into 8 fractions and dried by vacuum centrifugation.

To enrich Ksu peptides, tryptic peptides dissolved in NETN buffer (100 mM NaCl, 1 mM EDTA, 50 mM Tris-HCl, 0.5% (*v*/*v*) NP-40, pH 8.0) were incubated with pre-washed antibody beads (PTM Biolabs Inc, Hangzhou, China) at 4 °C overnight with gentle shaking. The beads were washed four times with NETN buffer and twice with ddH_2_O. The bound peptides were eluted from the beads with 0.1% trifluoroacetic acid (TFA). The eluted fractions were combined and vacuum-dried. The resulting peptides were cleaned with C18 ZipTips (Merk Millipore company, Danvers, MA, USA) according to the manufacturer’s instructions and analyzed by LC–MS/MS.

### 4.4. Proteomic Analysis by LC–MS/MS

Peptides were first dissolved in 0.1% (*w*/*v*) formic acid (FA) and directly loaded onto a reversed-phase pre-column (Acclaim PepMap 100, Thermo Fisher Scientific company, Waltham, MA, USA). Peptide separation was performed using a reversed-phase analytical column (Acclaim PepMap RSLC, Thermo Scientific). The gradient consisted of solvent B (0.1% FA in 98% ACN) increased from 7% to 22% for 24 min, from 22% to 35% for 8 min, at 80% for 5 min, and finally holding at 80% for the last 3 min; all increments were performed at a constant flow rate of 280 nL/min on an EASY-nLC 1000 UPLC system. The resulting peptides were analyzed using Q Exactive^TM^ with a hybrid quadrupole-Orbitrap mass spectrometer (Thermo Fisher Scientific company).

The peptides were subjected to a nanospray ionization (NSI) source before analysis with tandem mass spectrometry (MS/MS) in Q Exactive^TM^ plus (Thermo) coupled online to the UPLC. Intact peptides were detected in the Orbitrap at a resolution of 70,000. Peptides were selected for MS/MS with the normalized collision energy (NCE) set at 30; ion fragments were detected in the Orbitrap at a resolution of 17,500. A data-dependent procedure that alternated between one MS scan followed by 20 MS/MS scans was applied for the top 20 precursor ions above a threshold ion count of 5E3 in the MS survey scan with 15 s dynamic exclusion. The electrospray voltage applied was 2.0 kV. Automatic gain control (AGC) was used to prevent overfilling of the ion trap; 5E4 ions were accumulated for generating the MS/MS spectra. For MS scans, the *m*/*z* scan range was 350 to 1800.

### 4.5. Database Search

The resulting MS/MS data were processed using MaxQuant with an integrated Andromeda search engine (v.1.4.1.2). Tandem mass spectra were searched against the Uniprot_Oryza sativa database concatenated with a reverse decoy database. Trypsin/P was specified as the cleavage enzyme with up to 4 missing cleavages, 5 modifications per peptide, and 5 charges. Mass error was set to 10 ppm for precursor ions and 0.02 Da for fragment ions. Carbamidomethylation on Cys was specified as fixed modification, and oxidation on Met, succinylation on lysine, and acetylation on protein N termini were specified as variable modifications. False discovery rate (FDR) thresholds for protein, peptide, and modification site were specified at 1%. Minimum peptide length was set at 7 amino acids. All the other parameters in MaxQuant were set to default values. The site localization probability was set to >0.75.

### 4.6. Succinylated Protein Annotation Analysis

The GO annotation proteome was derived from the UniProt-GOA database. Identified protein IDs were first converted to UniProt IDs and then mapped to GO IDs using the protein IDs. If the identified proteins were not annotated by the UniProt-GOA database, the InterProScan (http://www.ebi.ac.uk/interpro/) software was used to annotate the protein’s GO domain functional description based on protein sequence alignment. The proteins were then classified with GO annotation (http://www.geneontology.org/) on the basis of three categories: biological process, cellular component, and molecular function.

InterProScan is a database that integrates diverse information regarding protein families, domains, and functional sites and provides Web-based interfaces and services free to the public. Central to the database are diagnostic models, known as signatures, against which protein sequences can be searched to determine their potential function. InterPro is useful for large-scale analysis of whole genomes and meta-genomes, as well as for characterizing individual protein sequences.

Kyoto Encyclopedia of Genes and Genomes (KEGG) connects known information on molecular interaction networks. The KEGG online KAAS tool was used to obtain the annotated protein’s KEGG database descriptions before mapping the annotation results onto the KEGG pathway database using the KEGG online KEGG mapper. The subcellular localization predication software Wolfpsort (http://wolfpsort.seq.cbrc.jp/) was used to predict proteins’ subcellular localization.

### 4.7. Functional Enrichment Analysis

The functional annotation tool, DAVID Bioinformatics Resources 6.7, was used to identify enriched GO terms, KEGG IDs, and domains. Fisher’s exact test (two tail test) was employed to test the enrichment of the protein-containing international protein index (IPI) entries against all IPI proteins. Correction for multiple hypothesis testing was carried out using standard false discovery rate control methods. Any term with a corrected *p*-value <0.05 was considered significant.

### 4.8. Succinylated Peptide Sequence Motif Discovery

Soft motif-x (http://motif-x.med.harvard.edu/motif-x.html) was used to analyze the sequence models constituted of amino acids in specific positions of modifier-21-mers (10 amino acids upstream and downstream of the succinylation site) for all protein sequences. All database protein sequences were used as background database parameters; other parameters were set at default.

### 4.9. Motif Logo-Based Clustering Analysis

All the lysine-succinylated substrate categories obtained after enrichment were collated along with their *p* values and filtered for the categories least enriched with clusters at *p* value <0.05. This filtered *p* value matrix was transformed with the function x = −log10 (*p* value). The x values were then z-transformed for each category. The z scores were clustered by one-way hierarchical clustering (Euclidean distance, average linkage clustering) in Genesis. Cluster membership was visualized by a heat map using the “heatmap.2” function from the “gplots” R-package.

## Supplementary Materials

The following are available online at https://www.mdpi.com/1422-0067/20/23/5911/s1.
