# Supplementary material for: Global Proteomic Analysis Reveals Widespread Lysine Succinylation in Rice Seedlings
Source: Int J Mol Sci. 2019 Nov 25;20(23):5911. doi: 10.3390/ijms20235911 (PMC6929033; doi:10.3390/ijms20235911)
Supplement: Supplementary file 1 [file ijms-20-05911-s001.zip › Supplementary Figures.docx]

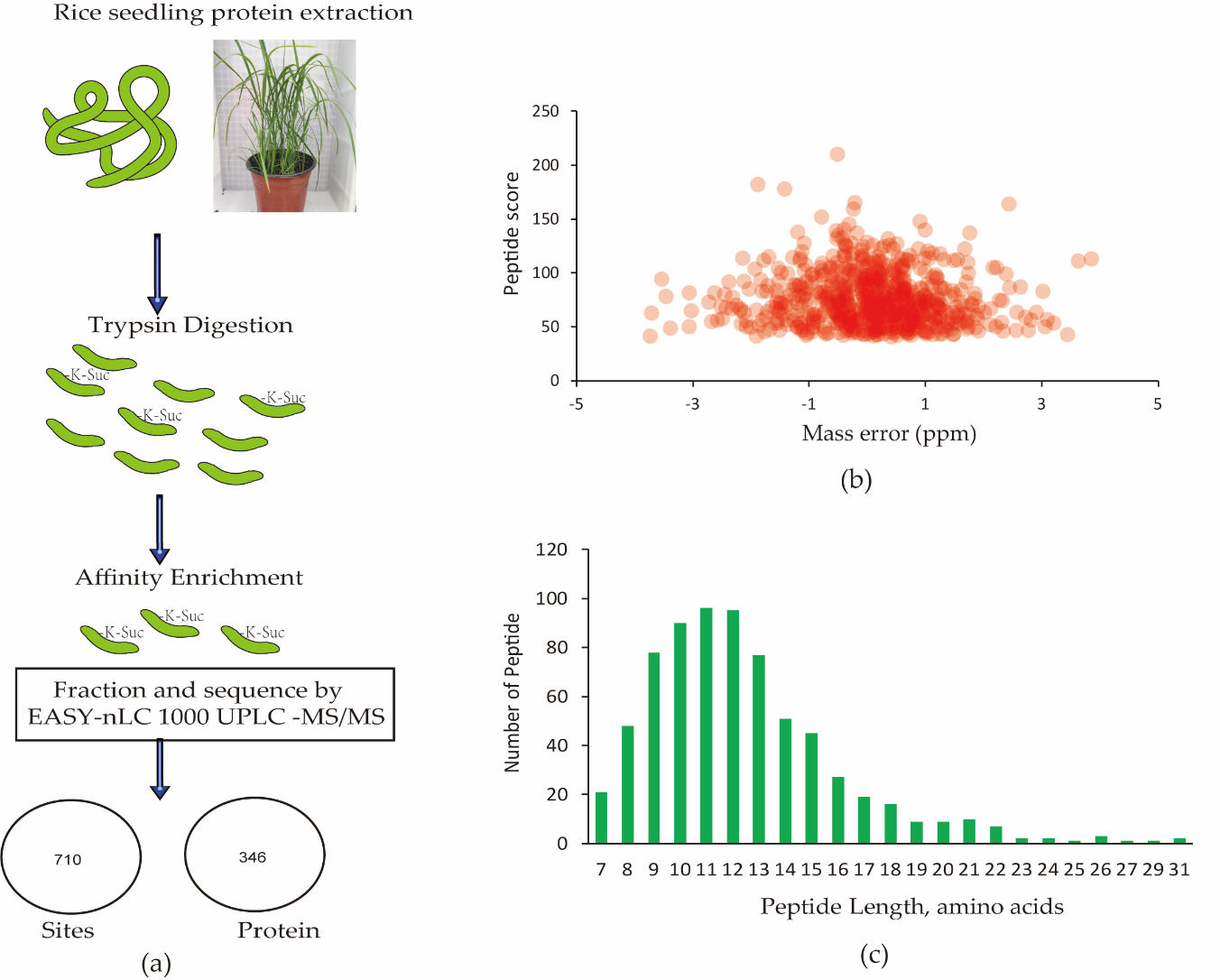


**Figure 1.** (**a**) Procedure for analyzing lysine succinylome in Oryza sativa. (**b**) Mass error distribution of all identified succinylated peptides. (**c**) Peptide length distribution.


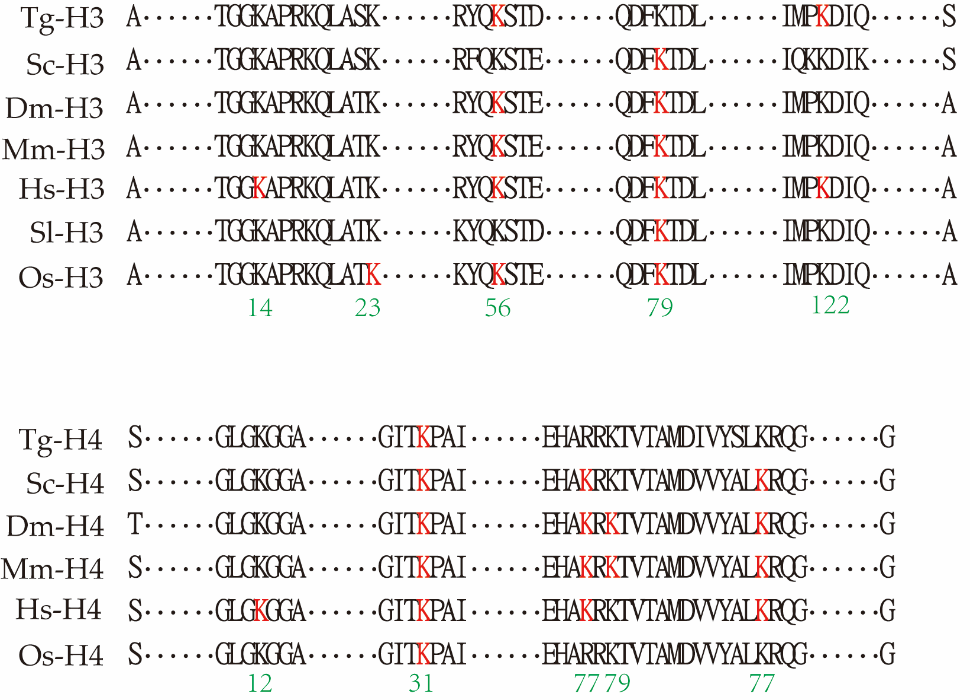


**Figure 2.** Comparison of the succinylated residues in rice histone H3 and H4 with other species. Red letters indicate succinylation sites. Numbers below the sequences represent the position of amino acid (Tg: *T. gondii*; Dm: *D. melanogaster*; Sc: *S. cerevisiae*; Mm: *M. musculus*; Hs: *H. sapiens*; Sl: *S. lycopersicum*; Os: *O. sativa*).


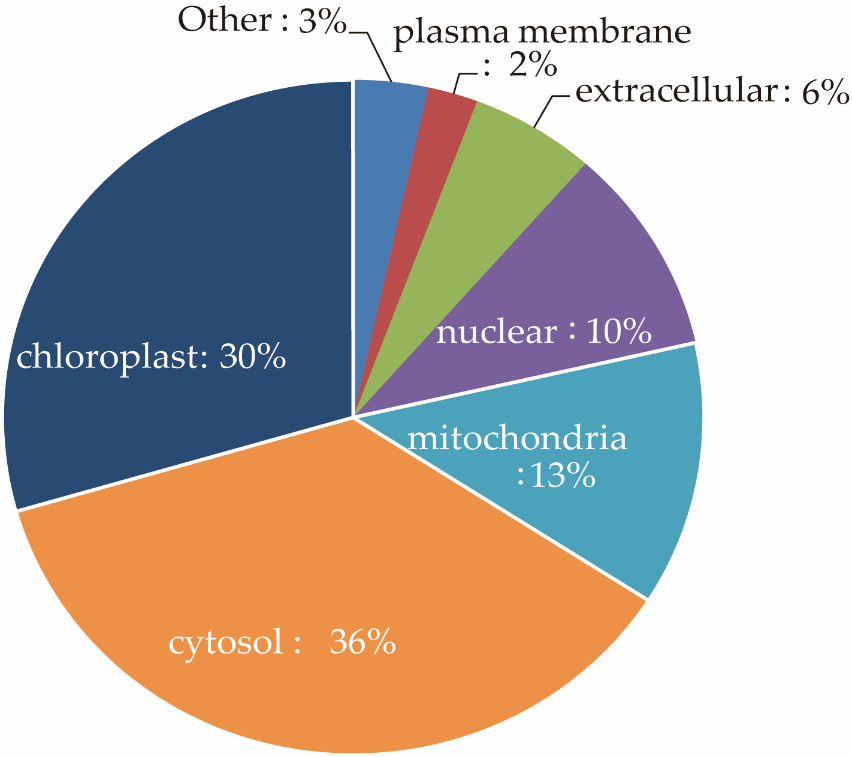


**Figure 3.** Subcellular localization of succinylated proteins identified in rice embryos.


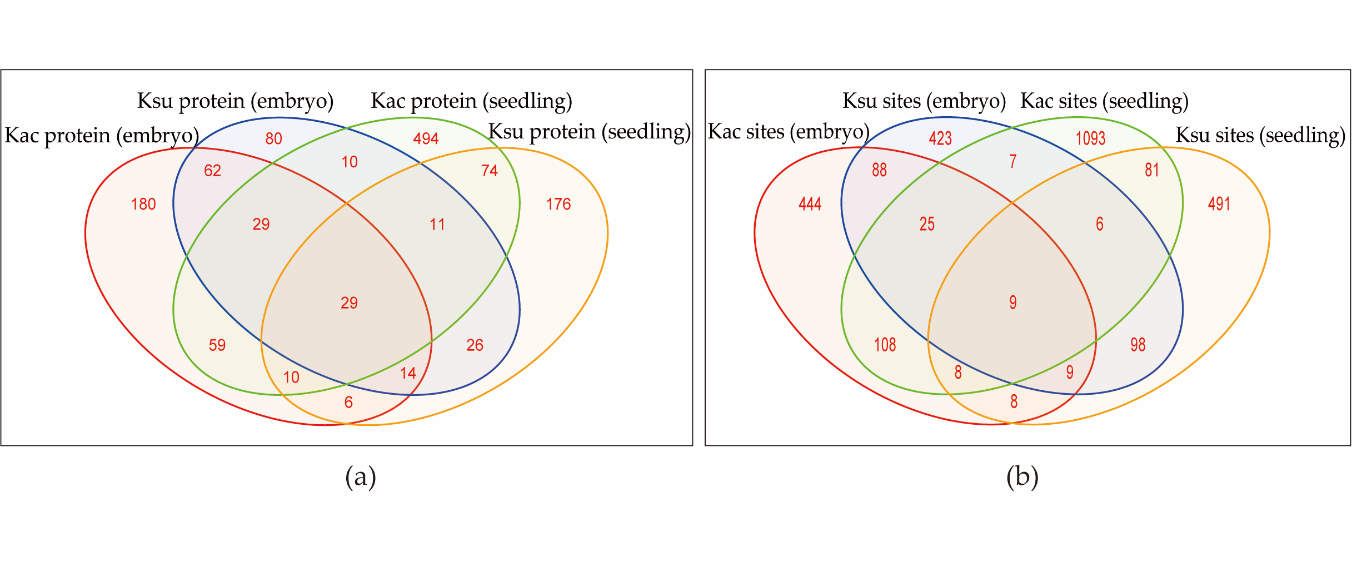


**Figure 4.** (a) Venn diagram of overlapping PTMs in rice embryos and seedlings. (b) Venn diagram of overlapping PTMs sites in rice embryos and seedlings.
